# Supplementary material for: Genome-wide identification, characterization and gene expression of BES1 transcription factor family in grapevine (Vitis vinifera L.)
Source: Sci Rep. 2023 Jan 5;13:240. doi: 10.1038/s41598-022-24407-y (PMC9816167; doi:10.1038/s41598-022-24407-y)
Supplement: Supplementary file 3 — Supplementary Information. [file 41598_2022_24407_MOESM3_ESM.zip › Vvi_Atr/Vitis_vinifera.PN40024.v4.dna_sm.toplevel.fa.vs.Amborella_trichopoda.AMTR1.0.dna_sm.toplevel.fa.html/Atr-AmTr_v1.0_scaffold00169.html]

|  |  |  |  |  |  |  |  |  |  |  |  |  |  |
| --- | --- | --- | --- | --- | --- | --- | --- | --- | --- | --- | --- | --- | --- |
| Duplication depth | Reference chromosome | Collinear blocks | | | | | | | | | | | |
| 0 | Atr-ERN10107 |  |  |  |  |  |  |  |  |  |  |  |  |
| 0 | Atr-ERN10108 |  |  |  |  |  |  |  |  |  |  |  |  |
| 0 | Atr-ERN10109 |  |  |  |  |  |  |  |  |  |  |  |  |
| 0 | Atr-ERN10110 |  |  |  |  |  |  |  |  |  |  |  |  |
| 0 | Atr-ERN10111 |  |  |  |  |  |  |  |  |  |  |  |  |
| 0 | Atr-ERN10112 |  |  |  |  |  |  |  |  |  |  |  |  |
| 0 | Atr-ERN10113 |  |  |  |  |  |  |  |  |  |  |  |  |
| 0 | Atr-ERN10114 |  |  |  |  |  |  |  |  |  |  |  |  |
| 0 | Atr-ERN10115 |  |  |  |  |  |  |  |  |  |  |  |  |
| 0 | Atr-ERN10116 |  |  |  |  |  |  |  |  |  |  |  |  |
| 0 | Atr-ERN10117 |  |  |  |  |  |  |  |  |  |  |  |  |
| 0 | Atr-ERN10118 |  |  |  |  |  |  |  |  |  |  |  |  |
| 0 | Atr-ERN10119 |  |  |  |  |  |  |  |  |  |  |  |  |
| 0 | Atr-ERN10120 |  |  |  |  |  |  |  |  |  |  |  |  |
| 0 | Atr-ERN10121 |  |  |  |  |  |  |  |  |  |  |  |  |
| 0 | Atr-ERN10122 |  |  |  |  |  |  |  |  |  |  |  |  |
| 0 | Atr-ERN10123 |  |  |  |  |  |  |  |  |  |  |  |  |
| 0 | Atr-ERN10124 |  |  |  |  |  |  |  |  |  |  |  |  |
| 0 | Atr-ERN10125 |  |  |  |  |  |  |  |  |  |  |  |  |
| 0 | Atr-ERN10126 |  |  |  |  |  |  |  |  |  |  |  |  |
| 0 | Atr-ERN10127 |  |  |  |  |  |  |  |  |  |  |  |  |
| 0 | Atr-ERN10128 |  |  |  |  |  |  |  |  |  |  |  |  |
| 0 | Atr-ERN10129 |  |  |  |  |  |  |  |  |  |  |  |  |
| 0 | Atr-ERN10130 |  |  |  |  |  |  |  |  |  |  |  |  |
| 0 | Atr-ERN10131 |  |  |  |  |  |  |  |  |  |  |  |  |
| 0 | Atr-ERN10132 |  |  |  |  |  |  |  |  |  |  |  |  |
| 0 | Atr-ERN10133 |  |  |  |  |  |  |  |  |  |  |  |  |
| 0 | Atr-ERN10134 |  |  |  |  |  |  |  |  |  |  |  |  |
| 0 | Atr-ERN10135 |  |  |  |  |  |  |  |  |  |  |  |  |
| 0 | Atr-ERN10136 |  |  |  |  |  |  |  |  |  |  |  |  |
| 0 | Atr-ERN10137 |  |  |  |  |  |  |  |  |  |  |  |  |
| 0 | Atr-ERN10138 |  |  |  |  |  |  |  |  |  |  |  |  |
| 0 | Atr-ERN10139 |  |  |  |  |  |  |  |  |  |  |  |  |
| 0 | Atr-ERN10140 |  |  |  |  |  |  |  |  |  |  |  |  |
| 0 | Atr-ERN10141 |  |  |  |  |  |  |  |  |  |  |  |  |
| 0 | Atr-ERN10142 |  |  |  |  |  |  |  |  |  |  |  |  |
| 0 | Atr-ERN10143 |  |  |  |  |  |  |  |  |  |  |  |  |
| 0 | Atr-ERN10144 |  |  |  |  |  |  |  |  |  |  |  |  |
| 0 | Atr-ERN10145 |  |  |  |  |  |  |  |  |  |  |  |  |
| 0 | Atr-ERN10146 |  |  |  |  |  |  |  |  |  |  |  |  |
| 0 | Atr-ERN10147 |  |  |  |  |  |  |  |  |  |  |  |  |
| 0 | Atr-ERN10148 |  |  |  |  |  |  |  |  |  |  |  |  |
| 0 | Atr-ERN10149 |  |  |  |  |  |  |  |  |  |  |  |  |
| 0 | Atr-ERN10150 |  |  |  |  |  |  |  |  |  |  |  |  |
| 0 | Atr-ERN10151 |  |  |  |  |  |  |  |  |  |  |  |  |
| 0 | Atr-ERN10152 |  |  |  |  |  |  |  |  |  |  |  |  |
